# Maternal serum retinol, vitamin K, and ferritin profiles during pregnancy, gestational diabetes mellitus, and offspring neurodevelopment: a prospective case-control study

**DOI:** 10.3389/fnut.2026.1967646

**Published:** 2026-10-23

**Authors:** Changke Jiang, Yulu Pang, Jinfeng Yu, Shiling Yan, Xiaoqing Liu, Tingyu Li

**Affiliations:** 1 Department of Neonatology, Chongqing Yongchuan District Maternal and Child Health Care Hospital, Chongqing, China; 2 Department of Child Health Care, Chongqing Yongchuan District Maternal and Child Health Care Hospital, Chongqing, China; 3 Department of Pediatrics, Chongqing Yongchuan District Maternal and Child Health Care Hospital, Chongqing, China; 4 Department of Child Health Care, Growth, Development and Mental Health Center of Children and Adolescents, Chongqing Key Laboratory of Child Neurodevelopment and Cognitive Disorders, National Clinical Research Center for Children and Adolescents' Health and Diseases, Ministry of Education Key Laboratory of Child Development and Disorders, Children's Hospital of Chongqing Medical University, Chongqing, China

**Keywords:** ferritin, gestational diabetes mellitus, maternal micronutrients, neurodevelopment, pregnancy, vitamin A, vitamin K

## Abstract

**Background:**

Gestational diabetes mellitus (GDM) is associated with adverse offspring neurodevelopment, but the role of maternal circulating biomarkers—specifically serum retinol, serum vitamin K, and serum ferritin—in relation to offspring neurodevelopmental performance remains unclear. This study examined trimester-specific levels of these biomarkers in GDM vs. participants without GDM and their associations with infant neurodevelopmental outcomes at 12 months.

**Methods:**

In this prospective case-control study, 240 mother-infant pairs who were enrolled during pregnancy, classified according to GDM status after OGTT screening, and followed through delivery and offspring assessment at 12 months. Maternal serum retinol, serum vitamin K, and serum ferritin were measured in each trimester. Offspring neurodevelopment was assessed using the Gesell Developmental Schedules, with suboptimal neurodevelopmental performance defined as Total Gesell Score < 85. Multivariable logistic regression models were adjusted for maternal age, pre-pregnancy BMI, years of formal education, gestational age at delivery, infant sex, and maternal HbA1c. Linear regression models included the same covariates together with maternal CRP and IL-6.

**Results:**

Participants with GDM had significantly lower serum retinol and serum vitamin K concentrations and higher serum ferritin concentrations across all trimesters compared with control participants (all *p* < 0.001). Offspring in the GDM group had lower Total Gesell Scores (90.4 vs. 95.9, *p* < 0.001) and higher prevalence of Total Gesell Score < 85 (72.5% vs. 31.7%, *p* < 0.001) compared with offspring in the control group. Although the mean differences were statistically significant, the group means remained within broadly similar interpretive ranges, and the < 85 threshold includes borderline scores. In fully adjusted logistic regression, higher serum ferritin was independently associated with increased odds of suboptimal neurodevelopmental performance (OR = 1.22 per 10 ng/mL, 95% CI: 1.004–1.34, *p* = 0.036), while vitamin A and K were not significant. An exploratory composite Nutritional Risk Score of 1, indicating the presence of any one of the three prespecified sample-specific quartile-defined biomarker categories (serum retinol in the lowest quartile, serum vitamin K in the lowest quartile, or serum ferritin in the highest quartile), was associated with a nearly five-fold higher odds of suboptimal neurodevelopmental performance (OR = 4.92, 95% CI: 1.65–14.64, *p* = 0.004; however, the association was not monotonic across higher score categories. Linear regression confirmed ferritin as a significant negative predictor of Total Gesell Score (β = −0.05, *p* = 0.003).

**Conclusions:**

In participants with GDM, higher maternal ferritin–but not vitamins A or K–is independently associated with offspring suboptimal neurodevelopmental performance. A simple composite nutritional risk score may help identify at-risk infants. These findings support monitoring ferritin and considering overall nutritional risk beyond glycaemic control in GDM care.

## Introduction

Gestational diabetes mellitus (GDM) is one of the most common metabolic complications of pregnancy and is characterized by glucose intolerance and insulin resistance which may disrupt maternal, placental, and fetal metabolic homeostasis (1–3). In addition to dysglycemia, GDM has been associated with chronic low-grade inflammation, oxidative stress, and broader metabolic dysregulation (4, 5). Compared with offspring of participants without GDM, offspring exposed to GDM have an increased risk of adverse perinatal and metabolic outcomes, including macrosomia and neonatal hypoglycemia, as well as adverse longer-term metabolic and neurodevelopmental outcomes (6). These associations provide a rationale for investigating maternal biological pathways that may coexist with GDM and contribute to variation in fetal and early postnatal development.

Maternal nutritional and metabolic status can influence fetal neurodevelopment through effects on placental nutrient transfer, substrate availability, oxidative balance, inflammation, neuronal differentiation, myelination, synaptogenesis, and other developmental processes (7, 8). Numerous dietary micronutrients are relevant to these pathways; however, the present study focused specifically on vitamin A, vitamin K, and iron for several reasons. First, each of these nutrients has been individually linked to either GDM risk and/or neurodevelopmental outcomes in human pregnancy studies: lower vitamin A levels have been associated with GDM in some populations and with impaired cognitive outcomes in children (9); and low iron status has been associated with impaired neurodevelopment, with serum ferritin, the primary biomarker of iron stores, linked to both GDM risk and adverse neurodevelopmental outcomes (10–12). Second, these nutrients play important roles in overlapping but partially distinct biological processes—vitamin A in neuronal differentiation and synaptogenesis, vitamin K in sphingolipid metabolism and myelination, and iron in oxygen transport, neurotransmitter synthesis, and myelination (5, 6)— with plausible relevance to both GDM-related metabolic dysregulation and fetal neurodevelopment. Third, the corresponding serum biomarkers (retinol, vitamin K, and ferritin) are measurable using established laboratory methods and are routinely available in clinical settings (11, 13). We specifically selected fat-soluble vitamins (A and K) because GDM is characterized by chronic low-grade inflammation and oxidative stress (4, 5). Fat-soluble vitamins are particularly vulnerable to oxidative depletion and have been implicated in the pathophysiology of GDM and its complications (5, 12). Vitamin A and K are stored in adipose tissue and the liver, and their metabolism may be altered in GDM due to insulin resistance and altered lipid metabolism (12, 14). Iron was included because of its dual role as an essential nutrient for neurodevelopment and as an acute-phase reactant whose interpretation is complicated by GDM-related inflammation (15, 16). While other nutrients, such as B vitamins, are also relevant to GDM and child neurodevelopment (5, 6), we selected these three because they represent both fat-soluble vitamins and a mineral, have well-established and routinely measured biomarkers, and are linked to GDM and neurodevelopment through overlapping but distinct biological pathways (12, 14). This selection was exploratory and not intended to be exhaustive.

Vitamin A is required for retinoid signaling and has important roles in neuronal differentiation, synaptic development, and visual system maturation (17). Human studies have also examined associations between maternal vitamin A status and pregnancy metabolic outcomes, including GDM, although the direction and magnitude of these associations remain incompletely established (14). Vitamin K, particularly phylloquinone, has been implicated in sphingolipid metabolism and other processes relevant to nervous-system development, while evidence regarding maternal vitamin K status in GDM remains comparatively limited (18). This relative paucity of pregnancy-specific evidence was one reason for examining serum vitamin K as an exploratory biomarker rather than assuming a clinically established relationship (19).

Iron is essential for fetal brain development because it contributes to cellular energy metabolism, neurotransmitter synthesis, and myelination (15). Maternal iron-related status may influence fetal iron endowment during sensitive developmental periods and thereby affect neurodevelopmental processes; however, maternal serum ferritin must be interpreted cautiously (20). Ferritin is a storage-related biomarker and an acute-phase reactant, and elevated concentrations during pregnancy may reflect inflammation, altered iron sequestration, metabolic dysfunction, increased iron stores, or a combination of these processes (8, 16). Accordingly, serum ferritin cannot by itself establish iron overload or functional iron excess (21). In pregnancy, serum ferritin levels are influenced by both iron stores and inflammatory status, requiring careful interpretation—particularly in GDM where inflammation is common (22). These considerations underscore the need to interpret ferritin alongside other measures of iron status when possible.

Despite the individual associations described above, no prior study has simultaneously tracked trimester-specific changes in serum retinol, serum vitamin K, and serum ferritin in participants with and without GDM, nor examined their combined associations with early neurodevelopment. Considering these biomarkers together is potentially informative because GDM does coexist with dysglycemia, inflammation, oxidative stress, and altered nutritional or iron-related physiology (5, 10, 12, 14, 23). The combination of these biomarkers may reveal patterns of nutritional and metabolic risk that are not apparent when examining each nutrient individually, as they may interact through shared metabolic pathways or have synergistic effects on fetal brain development (5, 6, 24). Their combined evaluation therefore provides an exploratory framework for assessing whether multiple sample-specific quartile-defined biomarker cutoffs identify patterns associated with offspring neurodevelopment beyond any single marker. However, the biological effects of these biomarkers should not be assumed to be additive, and the composite approach requires independent validation.

To address these gaps and build upon previous human evidence linking each biomarker individually to GDM and/or neurodevelopmental outcomes, this prospective case-control study investigated maternal serum retinol, serum vitamin K, and serum ferritin across all three trimesters in participants who subsequently met criteria for GDM and in control participants without GDM. We examined their associations with offspring neurodevelopmental performance at 12 months. Previous evidence links GDM to metabolic and inflammatory dysregulation, and vitamins A and K, as well as iron, have established biological roles in neurodevelopment. On this basis, we *a priori* hypothesized that maternal serum biomarker profiles would differ according to GDM status. We also hypothesized that these biomarkers might be associated with offspring neurodevelopmental performance after adjustment for relevant maternal and perinatal covariates, and that the association between maternal serum biomarkers and offspring neurodevelopment might differ according to GDM status (exploratory interaction analysis). We did not assume that GDM status and maternal biomarkers were independent causal factors; rather, we explored whether they might represent interrelated pathways reflecting broader metabolic and inflammatory disturbances that jointly influence fetal neurodevelopment. This conceptual framework guided our analytical approach, including the use of continuous maternal HbA1c rather than binary GDM status in the multivariable models to capture the full spectrum of glycemic exposure. We also explored a sample-specific composite biomarker-risk score. Given the exploratory nature of this approach, we did not assume that the effects of the three biomarkers would be additive or that the composite score would demonstrate a monotonic dose-response relationship.

## Methods

This prospective case-control study was conducted in accordance with the principles of the Declaration of Helsinki and the Strengthening the Reporting of Observational Studies in Epidemiology (STROBE) guidelines. The STROBE checklist was followed to ensure transparent and complete reporting of the study design, participant selection, data collection, and statistical analyses.

### Study design and setting

This was a prospective case-control study conducted at Chongqing Yongchuan District Maternal and Child Health Care Hospital between February 9, 2025, and May 3, 2026. Participants were enrolled during early pregnancy and classified according to GDM status following universal OGTT screening at 24–28 weeks of gestation. The study protocol was approved by the Institutional Review Board of Chongqing Yongchuan District Maternal and Child Health Care Hospital [approval number: (2025)1]. Written informed consent was obtained from all participating women prior to enrolment. For offspring neurodevelopmental assessments, separate written consent was obtained from mothers. All data were anonymized prior to analysis to protect participant confidentiality.

### Participants

Inclusion criteria were pregnant women aged 18–45 years with singleton pregnancies who received routine antenatal care at the participating hospital. Exclusion criteria were pre-existing type 1 or type 2 diabetes mellitus, chronic hypertension, thyroid disorders, autoimmune diseases, chronic infectious diseases, major fetal anomalies identified on prenatal ultrasound, multiple gestation (twins or higher), any condition that could independently affect maternal micronutrient status or fetal neurodevelopment (e.g., malabsorption syndromes, chronic liver or kidney disease), and inability or unwillingness to provide written informed consent (Figure 1).

**Figure 1:**
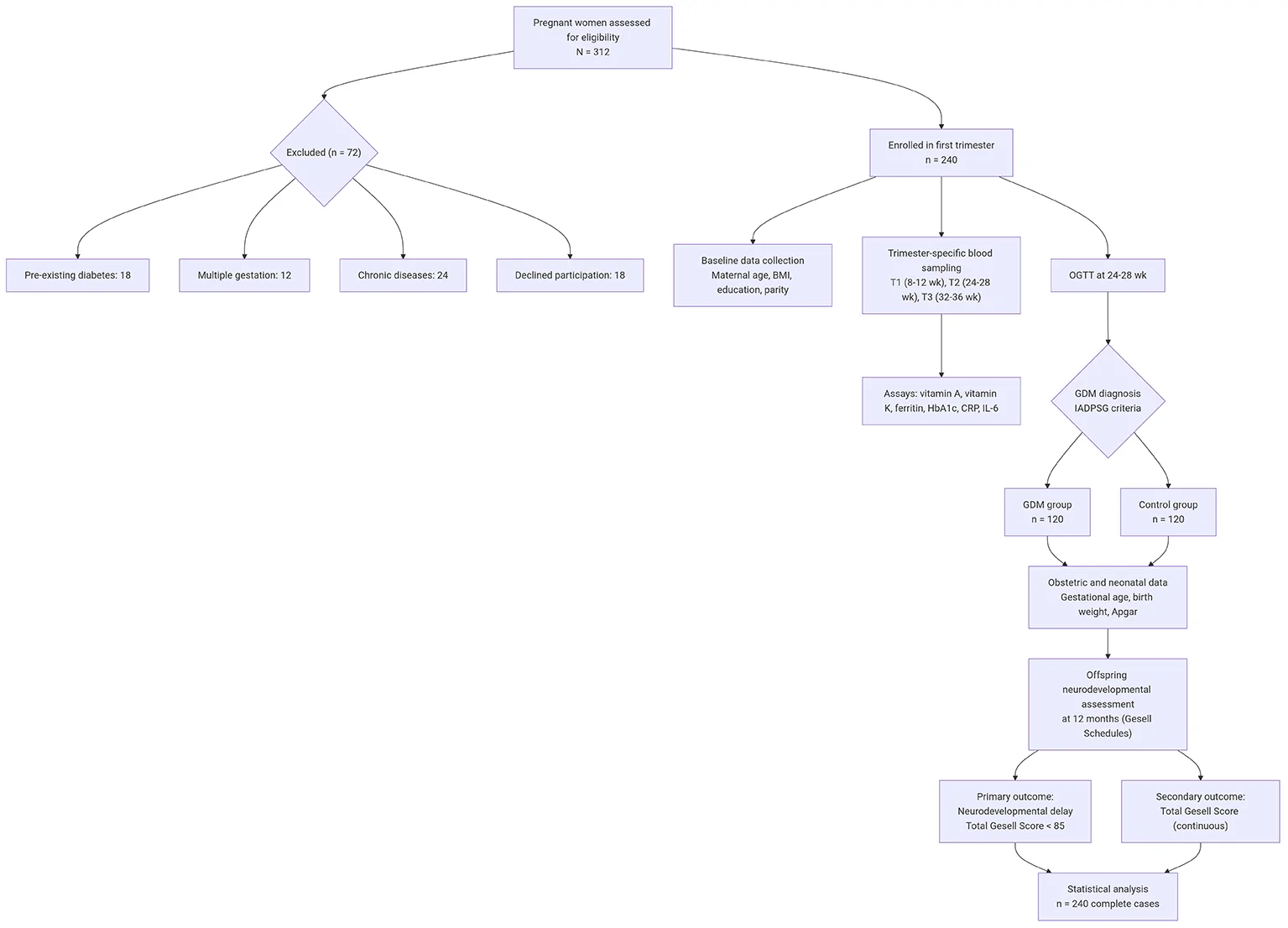
Flowchart of study.

### GDM diagnosis and group assignment

All enrolled women underwent universal screening for GDM between 24 and 28 weeks of gestation using a 75-g oral glucose tolerance test (OGTT) after a 10-h overnight fast. Venous plasma glucose concentrations were measured at fasting, 1 h, and 2 h post-glucose load. GDM was diagnosed at 24–28 weeks of gestation using the one-step 75-g oral glucose tolerance test strategy recommended by the International Association of Diabetes and Pregnancy Study Groups (IADPSG) and adopted by the American Diabetes Association Professional Practice Committee for Diabetes Standards of Care in Diabetes −2026 (25). Specifically, participants were diagnosed with GDM if any of the following thresholds were met: fasting plasma glucose ≥5.1 mmol/L (92 mg/dL), 1-h plasma glucose ≥10.0 mmol/L (180 mg/dL), or 2-h plasma glucose ≥8.5 mmol/L (153 mg/dL). Women meeting at least one criterion were assigned to the GDM group; those with all three values below the thresholds were assigned to the control group. GDM status served as the primary exposure variable for between-group comparisons.

### Sample size

A total of 240 mother–infant pairs (120 in the GDM group and 120 in the control group) were enrolled. Sample size was calculated based on an expected difference of 6 points in the mean Total Gesell Developmental Score between groups, with an assumed standard deviation of 10, 80% power, and a two-sided alpha level of 0.05. The expected difference of 6 points was derived from previous studies comparing neurodevelopmental outcomes in children of mothers with and without GDM, which have reported differences of 5–8 points on standardized developmental assessments at 12–24 months (6, 26). A 6-point difference on the Gesell DQ scale is considered clinically meaningful as it corresponds to approximately half a standard deviation and may influence the proportion of children falling below diagnostic thresholds for developmental delay. The sample size was increased by 20% to account for potential attrition and incomplete follow-up. This calculation was designed for the primary between-group comparison of the continuous neurodevelopmental score and was not specifically powered for each multivariable association involving serum retinol, serum vitamin K, or serum ferritin with the binary Total Gesell Score < 85 outcome. However, with 240 participants and 87 events (Total Gesell Score < 85), the study provides adequate power to examine the independent associations of the three biomarkers in logistic regression models, as the number of events exceeds the recommended minimum of 10 events per predictor variable (27) for our primary models (which include 6–8 covariates).

### Data collection procedures

Data were collected at multiple time points across pregnancy and the postpartum follow-up period.

### Baseline maternal data (first trimester)

At enrolment (gestational weeks 8–12), maternal demographic and clinical characteristics were obtained through structured interviews and medical record abstraction, including age (years), pre-pregnancy body mass index (BMI, kg/m^2^; calculated from self-reported pre-pregnancy weight and height measured at enrolment), years of formal education completed, parity (number of previous pregnancies delivered beyond 20 weeks of gestation), previous GDM history, and other relevant maternal medical history.

### Trimester-specific blood sampling

Maternal fasting venous blood samples (10 mL) were collected at three time points corresponding to the three trimesters of pregnancy: first trimester (8–12 weeks), second trimester (24–28 weeks, coinciding with OGTT), and third trimester (32–36 weeks). Maternal HbA1c was measured at the time of OGTT (24–28 weeks) and in the third trimester. Blood samples were collected into serum separator tubes, allowed to clot at room temperature for 30 min, and centrifuged at 3,000 rpm for 10 min within 2 h of collection. Serum aliquots were stored at −80 °C until analysis. All assays were performed in duplicate, and laboratory personnel were blinded to GDM status.

### Biochemical assays

Maternal serum retinol, serum vitamin K (phylloquinone), and serum ferritin concentrations were measured using validated standard laboratory methods. Serum retinol concentrations were quantified with a limit of detection of 0.05 μmol/L and intra- and inter-assay coefficients of variation < 8% (28, 29). Serum vitamin K concentrations were measured with a limit of detection of 0.05 ng/mL and intra- and inter-assay coefficients of variation < 10% (30). Serum ferritin concentrations were measured using a commercially available enzyme-linked immunosorbent assay (ELISA) kit (Sigma-Aldrich, Catalog No. RAB0197-1KT/SE120155). The assay was performed according to the manufacturer's instructions, with a sensitivity of 1.0 ng/mL and intra- and inter-assay coefficients of variation < 8%. Because assay-specific pregnancy validation data were not available for this platform, and because serum ferritin is influenced by both iron status and inflammation, we interpreted serum ferritin as a non-specific circulating biomarker that may reflect iron status, inflammation, or both, rather than as a definitive measure of total body iron or functional iron availability (31). This conservative interpretation acknowledges that the assay's performance characteristics may differ in pregnant women and that complementary measures of iron status (e.g., transferrin saturation, hepcidin) were not available. All assays were performed in duplicate, and laboratory personnel were blinded to GDM status.

Other Biochemical Markers: Fasting plasma glucose was measured using the glucose oxidase method. HbA1c was measured by high-performance liquid chromatography using a standardized NGSP-certified method. High-sensitivity C-reactive protein (CRP) and interleukin-6 (IL-6) were measured using commercially available ELISA kits. Throughout this manuscript, maternal HbA1c, maternal CRP, and maternal IL-6 refer to maternal serum measures.

Trimester-specific biomarker concentrations were used for descriptive comparisons between participants with GDM and control participants. For the primary analyses examining the relationship between maternal biomarker concentrations and offspring neurodevelopmental outcomes, the arithmetic mean of the three trimester-specific measurements was calculated for each participant and used as a summary exposure measure (*a priori*). As a *post-hoc* supplementary analysis to evaluate the appropriateness of this averaging approach, we examined within-individual changes using repeated-measures models (Results and Supplementary Table S1). We acknowledge that established reference intervals exist and are routinely used during pregnancy to diagnose individual vitamin deficiencies and iron overload. However, to our knowledge, no validated reference intervals exist for a combined three-biomarker classification in pregnancy, and no established precedent defines thresholds for such a combined classification. We therefore used an exploratory, distribution-based approach: the 25th and 75th percentiles of the participant-level trimester-averaged biomarker distributions in the full cohort were used to define lowest-quartile and highest-quartile categories for serum retinol, serum vitamin K, and serum ferritin, respectively. This approach is commonly used in nutritional epidemiology and biomarker studies for exploratory analyses when established clinical thresholds are not available for the specific analytical purpose (32, 33). These sample-specific categories are exploratory and are not intended to replace established clinical reference intervals for diagnosing deficiency or excess; they represent relative positions within the study population and should not be interpreted as clinical diagnostic criteria for vitamin A deficiency, vitamin K deficiency, iron deficiency, iron overload, or other disease states. The Nutritional Risk Score was then calculated by assigning one point for each prespecified biomarker-risk category.

### Dietary assessment

Maternal dietary intake was assessed at enrolment (first trimester, gestational weeks 8–12) using a validated semi-quantitative food frequency questionnaire (FFQ) adapted for the Chinese population (34). The FFQ consisted of 78 food items grouped into categories: cereals and grains, vegetables, fruits, meat and poultry, fish and seafood, eggs, dairy products, legumes, nuts, oils and fats, beverages, and snacks. For each food item, participants reported the frequency of consumption (daily, weekly, monthly, or rarely) and the portion size (using standard household measures, e.g., bowls, cups, pieces). Daily nutrient and energy intakes were calculated using the Chinese Food Composition Database (35). Total energy intake (kcal/day), macronutrients (carbohydrate, protein, fat in g/day), dietary fiber (g/day), and intakes of fruits & vegetables (g/day) and whole grains (g/day) were derived. All FFQs were administered by trained research assistants during a face-to-face interview.

### Obstetric and neonatal data

Information on gestational age at delivery (weeks), infant sex, birth weight (g), and 5-min Apgar score was abstracted from medical records following delivery.

### Offspring neurodevelopmental assessment (12 months)

Offspring neurodevelopmental outcomes were assessed at 12 months of age (±2 weeks) by trained developmental pediatricians blinded to maternal GDM status and biomarker results. The Gesell Developmental Schedules (GDDS), Chinese standardized version, was used as the primary assessment tool (36). The GDDS is designed to assess developmental functioning in children from birth to 6 years of age and has been standardized for the Chinese population with established age-specific national norms (37, 38).

The GDDS assesses child development across five functional domains: gross motor (large muscle movements including sitting, walking, and jumping), fine motor (fine hand and finger movements including grasping and manipulation), language (receptive and expressive language abilities), adaptive behavior (problem-solving, environmental adaptation, and cognitive flexibility), and personal-social behavior (social interaction, self-care, and interpersonal skills). For each domain, raw scores were converted to developmental quotients (DQs) using the formula DQ = (Developmental Age/Chronological Age) × 100, based on Chinese normative data. A Total Gesell Score was calculated as the mean of the five domain-specific DQ scores. For the primary binary analysis, a Total Gesell Score < 85 was used as a prespecified study-defined threshold for suboptimal neurodevelopmental performance. This threshold was selected for several reasons: (1) it aligns with Chinese clinical practice guidelines (37, 38) for early identification of infants at risk for developmental delay who may benefit from referral and early intervention; (2) as a more inclusive threshold, it increases sensitivity for detecting infants who may benefit from closer monitoring; and (3) it provides greater statistical power for examining associations with maternal biomarkers in our exploratory analyses. This threshold was intended for early identification and surveillance purposes rather than as a definitive clinical diagnosis of developmental delay.

We acknowledge that alternative GDDS score classifications exist in the literature, with some studies defining mild developmental delay as a DQ ≤ 75 and classifying scores between 75 and 85 as borderline (39–42). However, the < 85 threshold used in our study was intentionally chosen as a study-defined operational definition rather than a definitive clinical diagnosis of global developmental delay. Children with Total Gesell Scores ≥85 were classified as not meeting the study's operational threshold, recognizing that scores between 75 and 85 may represent borderline performance in alternative classification systems (37).

The primary outcome was suboptimal neurodevelopmental performance (binary) defined as Total Gesell Score < 85 at 12 months. Domain-specific DQ scores (gross motor, fine motor, language, adaptive, social) were examined as secondary outcomes.

### Statistical analysis

All statistical analyses were performed using Stata version 18.0 (StataCorp, College Station, TX, USA). Missing data for each variable were summarized. The overall missing rate was < 5% for all variables. For the primary analyses, complete case analysis was used; in sensitivity analyses, multiple imputation was performed to address potential bias from missing data. A two-tailed *p*-value < 0.05 was considered statistically significant. Continuous variables were tested for normality using the Shapiro-Wilk test. Normally distributed variables were summarized as mean ± standard deviation (SD) and compared between groups using independent Student's *t*-tests (43). Non-normally distributed variables (CRP, IL-6) were summarized as median and compared using the Mann-Whitney U test. Categorical variables were presented as frequencies (n, %) and compared using Pearson's chi-squared test or Fisher's exact test as appropriate. Baseline characteristics, glycemic and inflammatory markers, neonatal outcomes, and trimester-specific biomarker levels were compared between GDM and control groups using the appropriate parametric or non-parametric tests. Neurodevelopmental domain scores were compared using *t*-tests, and the prevalence of suboptimal neurodevelopmental performance was compared using chi-squared tests.

The primary adjustment set was prespecified before the main regression analyses based on clinical relevance, prior literature, and our conceptual framework in which maternal GDM status and maternal biomarker concentrations were hypothesized to represent interrelated pathways reflecting broader metabolic and inflammatory disturbances that jointly influence fetal neurodevelopment (4, 5). The prespecified primary adjustment set included maternal age, pre-pregnancy BMI, years of formal education, gestational age at delivery, infant sex, and maternal HbA1c. Multivariable logistic regression was used to examine the independent associations of mean maternal serum retinol, serum vitamin K, and serum ferritin concentrations with suboptimal neurodevelopmental performance, adjusted for this prespecified set. Maternal HbA1c was included as a continuous measure of glycemic exposure to account for variation in glycemic status across the full cohort. In sensitivity analyses, we also examined models using GDM status instead of HbA1c; the results are presented in Supplementary Table S3.

Gestational age at delivery was retained in the prespecified adjustment set because it is a well-established clinical confounder for offspring neurodevelopmental outcomes. In contrast, birth weight and 5-minute Apgar score were not included in the primary adjustment set because they are not established confounders but may represent intermediate or downstream perinatal characteristics on the causal pathway between maternal exposures and offspring neurodevelopment (44, 45). In particular, birth weight may lie on the pathway between maternal metabolic or nutritional exposures and subsequent offspring neurodevelopment, such that adjustment could result in overadjustment. In exploratory analyses, we examined whether birth weight and Apgar score mediated the relationship between GDM/biomarkers and neurodevelopment; these results are presented in Supplementary Table S4. This distinction is based on the conceptual role of each variable in the causal framework, not solely on temporal ordering.

Parity was considered during covariate assessment but was not included in the final primary models because it was not part of the prespecified adjustment set. In sensitivity analyses, the inclusion of parity did not materially alter the estimates of the primary associations, and it was therefore not retained in the final parsimonious models. Years of formal education was retained because it was prespecified as a sociodemographic confounder related to maternal health behaviors, access to care, and the child's developmental environment. Previous GDM history was also assessed as part of the maternal medical history; however, no participant reported a previous history of GDM, precluding its inclusion as a covariate.

CRP and IL-6 were included only in the linear regression models for the continuous Total Gesell Score, not in the primary logistic regression model, because inflammation is a central mechanism linking GDM to adverse neurodevelopmental outcomes and because serum ferritin is influenced by inflammation; they were excluded from the logistic model to avoid overfitting given the number of outcome events (*n* = 87 with Total Gesell Score < 85). Adjusted odds ratios (ORs) with 95% confidence intervals (CIs) were reported, and variance inflation factors were calculated to assess multicollinearity (VIF < 5 considered acceptable); all VIFs were < 1.5 (HbA1c: 1.44; ferritin: 1.35), confirming no problematic multicollinearity (46). In exploratory analyses, we examined whether GDM status modified the association between maternal serum ferritin and offspring neurodevelopment by including a Ferritin × GDM interaction term in both the logistic and linear regression models. These interaction analyses were adjusted for the same covariates as the primary models and are presented in Supplementary Table S5.

To evaluate the dose-response relationship between cumulative nutritional risk and suboptimal neurodevelopmental performance, the Nutritional Risk Score (0–3) was entered into logistic regression models as a categorical variable with score 0 as the reference category. A test for linear trend was conducted by including the score as a continuous variable in the same adjusted model, and marginal effects were estimated to calculate predicted probabilities of suboptimal neurodevelopmental performance at each risk score level.

Multivariable linear regression was fitted to assess the relationship between mean maternal serum retinol, serum vitamin K, and serum ferritin concentrations and Total Gesell Score as a continuous outcome, using the same covariate adjustment set together with maternal CRP and IL-6. Regression coefficients (β) with standard errors (SEs) and *p*-values were reported, and residual plots were examined to verify linear regression assumptions (linearity, normality of residuals, homoscedasticity).

To address the potential confounding effect of inflammation on ferritin levels, we conducted sensitivity analyses using CRP-adjusted ferritin values. Following an adaptation of the BRINDA regression correction approach (47), we regressed log-transformed ferritin on log-transformed CRP and used the residuals to calculate adjusted ferritin values. These adjusted ferritin values were then used as predictors in the logistic and linear regression models. A full BRINDA adjustment would require both CRP and α1-acid glycoprotein (AGP) measurements; however, AGP was not available in our dataset, and thus only CRP-adjusted ferritin could be examined. The results of these sensitivity analyses are presented in Supplementary Table S2.

## Results

### Study population

A total of 240 mother–infant pairs (120 with GDM and 120 without GDM) were included in the final analysis. Baseline characteristics of the study population are presented in Table 1. Women with GDM had significantly higher pre-pregnancy body mass index (26.93 ± 4.52 vs. 25.13 ± 3.44 kg/m^2^, *p* = 0.001) and delivered at an earlier gestational age (38.42 ± 1.69 vs. 38.92 ± 1.21 weeks, *p* = 0.009) compared with controls. No significant differences were observed between the two groups in maternal age, years of formal education, parity, or infant sex. In contrast, offspring of participants with GDM had lower birth weight and lower 5-min Apgar scores than offspring of control participants (both *p* ≤ 0.001). As expected, fasting glucose (103.73 ± 12.30 vs. 87.34 ± 9.14 mg/dL, *p* < 0.001) and maternal HbA1c (6.09 ± 0.63 vs. 5.22 ± 0.37%, *p* < 0.001) were substantially higher in the GDM group. Inflammatory markers, including maternal CRP (4.26 ± 2.49 vs. 2.97 ± 2.32 mg/L, *p* < 0.001) and maternal IL-6 (3.93 ± 1.58 vs. 2.18 ± 1.10 pg/mL, *p* < 0.001), were also significantly elevated in Participants with GDM.

**Table 1:** Maternal, perinatal, and dietary characteristics according to gestational diabetes mellitus status.

Variable	Control (*n* = 120)	GDM (*n* = 120)	*p*-value
Maternal age (years)	30.16 ± 5.41	30.12 ± 4.72	0.947
Pre-pregnancy BMI (kg/m^2^)	25.13 ± 3.44	26.93 ± 4.52	0.001
Education (years)	14.21 ± 2.88	13.92 ± 3.22	0.460
Gestational age at delivery (weeks)	38.92 ± 1.21	38.42 ± 1.69	0.009
Infant sex (male %)	51.7	51.8	0.985
Fasting glucose (mg/dL)	87.34 ± 9.14	103.73 ± 12.30	< 0.001
Maternal HbA1c (%)	5.22 ± 0.37	6.09 ± 0.63	< 0.001
Maternal CRP (mg/L)	2.97 ± 2.32	4.26 ± 2.49	< 0.001
Maternal IL6 (pg/mL)	2.18 ± 1.10	3.93 ± 1.58	< 0.001
Birth weight (g)	3,343 ± 363	3,187 ± 382	0.001
Apgar_5 min	9.22 ± 0.45	8.84 ± 0.51	< 0.001
Dietary intake			
Energy intake (kcal/day)	2,025.84 ± 75.04	2,114.14 ± 375.07	0.008
Carbohydrate (g/day)	237.80 ± 14.10	246.59 ± 29.80	0.004
Protein (g/day)	87.10 ± 4.10	85.53 ± 5.19	0.004
Fat (g/day)	70.10 ± 4.80	74.88 ± 14.04	0.003
Fiber (g/day)	25.60 ± 5.20	23.97 ± 7.52	0.047
Fruits and vegetables (g/day)	423.95 ± 61.10	383.15 ± 143.71	0.005
Whole grains (g/day)	146.20 ± 30.70	136.56 ± 45.46	0.054
Dietary vitamin A (μg RAE/day)	788.61 ± 115.16	712.58 ± 265.87	0.004
Dietary vitamin K (μg/day)	105.66 ± 21.64	98.74 ± 30.97	0.046
Dietary iron (mg/day)	18.24 ± 2.86	17.94 ± 3.25	0.439

Values are presented as mean ± SD unless otherwise indicated. Maternal CRP and maternal IL-6 were compared using the Mann–Whitney U test; other continuous variables were compared using independent-samples *t*-tests after assessment of distributional assumptions. Categorical variables were compared using Pearson's chi-square test or Fisher's exact test, as appropriate. BMI, body mass index; CRP, C-reactive protein; GDM, gestational diabetes mellitus; HbA1c, glycated hemoglobin; IL-6, interleukin-6.

Regarding dietary intake, women with GDM had significantly higher energy intake (2114.14 ± 375.07 vs. 2025.84 ± 75.04 kcal/day, *p* = 0.008), higher carbohydrate intake (246.59 ± 29.80 vs. 237.80 ± 14.10 g/day, p = 0.004), and higher fat intake (74.88 ± 14.04 vs. 70.10 ± 4.80 g/day, *p* = 0.003) compared with controls. Protein intake was lower in the GDM group (85.53 ± 5.19 vs. 87.10 ± 4.10 g/day, *p* = 0.004), as was dietary fiber intake (23.97 ± 7.52 vs. 25.60 ± 5.20 g/day, *p* = 0.047). Intake of fruits and vegetables was substantially lower in the GDM group (383.15 ± 143.71 vs. 423.95 ± 61.10 g/day, *p* = 0.005), while whole grain intake showed a trend toward being lower (136.56 ± 45.46 vs. 146.20 ± 30.70 g/day, *p* = 0.054).

With respect to specific micronutrient intakes, women with GDM had significantly lower dietary vitamin A intake (712.58 ± 265.87 vs. 788.61 ± 115.16 μg RAE/day, *p* = 0.004) and significantly lower dietary vitamin K intake (98.74 ± 30.97 vs. 105.66 ± 21.64 μg/day, *p* = 0.046) compared with controls. Dietary iron intake did not differ significantly between groups (17.94 ± 3.25 vs. 18.24 ± 2.86 mg/day, *p* = 0.439).

Offspring of mothers with GDM had lower birth weight (3,187 ± 382 vs. 3,343 ± 363 g, p = 0.001) and lower 5-min Apgar scores (8.84 ± 0.51 vs. 9.22 ± 0.45, *p* < 0.001) compared with controls.

### Maternal micronutrient levels across trimesters

Table 2 summarizes maternal serum vitamin A, vitamin K, and ferritin concentrations measured in each trimester. Throughout pregnancy, women with GDM had consistently lower **serum** retinol concentrations (first trimester: 0.34 ± 0.08 vs. 0.45 ± 0.08 μmol/L; second trimester: 0.36 ± 0.08 vs. 0.45 ± 0.08 μmol/L; third trimester: 0.36 ± 0.08 vs. 0.45 ± 0.07 μmol/L; all *p* < 0.001) and lower serum vitamin K concentrations (first trimester: 0.57 ± 0.09 vs. 0.72 ± 0.10; second trimester: 0.57 ± 0.10 vs. 0.70 ± 0.11; third trimester: 0.56 ± 0.10 vs. 0.72 ± 0.10; all *p* < 0.001) compared with control participants. Conversely, serum ferritin concentrations were significantly higher in the GDM group at all three time points (first trimester: 122.95 ± 30.85 vs. 77.33 ± 26.74 ng/mL; second trimester: 118.97 ± 29.79 vs. 80.70 ± 28.57 ng/mL; third trimester: 119.70 ± 31.45 vs. 83.37 ± 28.39 ng/mL; all *p* < 0.001).

**Table 2:** Maternal serum biomarker concentrations across three pregnancy timepoints according to gestational diabetes mellitus status.

Biomarker	Control (*n* = 120)	GDM (*n* = 120)	*p*-value
Serum retinol, T1 (μmol/L)	0.45 ± 0.08	0.34 ± 0.08	< 0.001
Serum retinol, T2 (μmol/L)	0.45 ± 0.08	0.36 ± 0.08	< 0.001
Serum retinol, T3 (μmol/L)	0.45 ± 0.07	0.36 ± 0.08	< 0.001
Serum vitamin K, T1 (ng/mL)	0.72 ± 0.10	0.57 ± 0.09	< 0.001
Serum vitamin K, T2 (ng/mL)	0.70 ± 0.11	0.57 ± 0.10	< 0.001
Serum vitamin K, T3 (ng/mL)	0.72 ± 0.10	0.56 ± 0.10	< 0.001
Serum ferritin, T1 (ng/mL)	77.33 ± 26.74	122.95 ± 30.85	< 0.001
Serum ferritin, T2 (ng/mL)	80.70 ± 28.57	118.97 ± 29.79	< 0.001
Serum ferritin, T3 (ng/mL)	83.37 ± 28.39	119.70 ± 31.45	< 0.001

Values are presented as mean ± SD. *P*-values were derived from independent-samples *t*-tests. T1, first trimester (8–12 weeks); T2, second trimester (24–28 weeks); T3, third trimester (32–36 weeks). Group-by-time interaction *p-*values from repeated-measures analyses were 0.517 for serum retinol, 0.249 for serum vitamin K, and 0.186 for serum ferritin. Complete repeated-measures results are provided in Supplementary Table S1.

We acknowledge that established reference intervals exist and are routinely used during pregnancy to diagnose individual vitamin deficiencies and iron overload. However, validated reference intervals for a combined three-biomarker classification in pregnancy are not available. In the full cohort, the lowest-quartile and highest-quartile thresholds were ≤ 0.35 μmol/L for serum retinol, ≤ 0.57 ng/mL for serum vitamin K, and ≥120.43 ng/mL for serum ferritin. These sample-specific thresholds describe the distribution of the study population and were used only for exploratory exposure classification. They should not be interpreted as clinical diagnostic thresholds for deficiency or excess. These exploratory thresholds require external validation.

To examine within-individual changes in biomarker concentrations over time, we performed repeated measures ANOVA with group (GDM vs. control) as the between-subjects factor and trimester as the within-subjects factor. For serum ferritin, the control group showed a slight increase over time (from 77.33 ± 26.74 ng/mL in the first trimester to 83.37 ± 28.39 ng/mL in the third trimester), while the GDM group showed a slight decrease (from 122.95 ± 30.85 ng/mL to 119.70 ± 31.45 ng/mL). However, the group-by-time interaction was not statistically significant [χ^2^(2) = 3.36, *p* = 0.186]. For serum retinol, levels remained stable over time in both groups, with no significant group-by-time interaction [χ^2^(2) = 1.32, *p* = 0.51]. Similarly, serum vitamin K levels showed no significant group-by-time interaction [χ^2^(2) = 2.78, *p* = 0.249]. The complete results are presented in Supplementary Table S1. These *post-hoc* supplementary repeated-measures analyses were conducted to assess the robustness of the trimester-averaging approach and were not part of the *a priori* primary outcome analyses. In sensitivity analyses using trimester-specific values in regression models, the results were consistent with those using mean values (data not shown).

### Offspring neurodevelopmental outcomes

Neurodevelopmental outcomes at 12 months of age are shown in Table 3. Children born to mothers with GDM scored significantly lower across all five Gesell developmental domains compared with controls. The mean Total Gesell Score was 90.4 ± 4.1 in the GDM group vs. 95.9 ± 5.1 in the control group (*p* < 0.001). The prevalence of participants meeting the study-defined operational threshold of Total Gesell Score < 85 was substantially higher among offspring of participants with GDM (72.5%, 87/120) than among offspring of control participants (31.7%, 38/120), with a chi-squared *p*-value of < 0.001.

**Table 3:** Offspring neurodevelopmental performance at 12 months according to maternal gestational diabetes mellitus status.

Domain	Control (*n* = 120) mean ±SD	GDM (*n* = 120) mean ±SD	*p*-value
Gross motor	97.0 ± 11.1	91.5 ± 10.1	< 0.001
Fine motor	94.6 ± 12.3	88.0 ± 9.8	< 0.001
Language	96.0 ± 11.1	88.5 ± 10.1	< 0.001
Adaptive	94.9 ± 10.8	90.4 ± 9.1	< 0.001
Social	97.2 ± 11.2	93.4 ± 8.8	0.005
Total Gesell Score	95.9 ± 5.1	90.4 ± 4.1	< 0.001
Total Gesell Score < 85, *n* (%)	38 (31.7%)	87 (72.5%)	< 0.001

Values for continuous outcomes are presented as mean ± SD and were compared using independent-samples *t*-tests. The binary outcomes were compared using Pearson's chi-square test. A Total Gesell Score < 85 was used as the study's prespecified study-defined threshold and should not be interpreted as a clinical diagnosis of developmental delay. Alternative classification systems in the literature may define mild developmental delay as DQ ≤ 75, with scores 75–85 classified as borderline (21–24).

### Association of maternal serum biomarker concentrations with suboptimal neurodevelopmental performance

Multivariable logistic regression was performed to examine the independent associations of mean trimester-averaged maternal serum biomarker concentrations with suboptimal neurodevelopmental performance, adjusting for maternal age, pre-pregnancy BMI, education years, gestational age at delivery, infant sex, and maternal HbA1c (Table 4). Higher mean serum ferritin was independently associated with increased odds of suboptimal neurodevelopmental performance (OR = 1.22 per 10 ng/mL, 95% CI: 1.004–1.34, *p* = 0.036). Neither mean serum retinol (OR = 0.02, 95% CI: 0.01–1.27, *p* = 0.238) nor mean serum vitamin K (OR = 1.15, 95% CI: 0.16–8.1, *p* = 0.260) reached statistical significance in the fully adjusted model. Among covariates, older maternal age (OR = 1.06, 95% CI: 1.01–1.13, *p* = 0.049), earlier gestational age at delivery (OR = 0.80, 95% CI: 0.64–0.99, *p* = 0.039), and male infant sex (OR = 1.98, 95% CI: 1.07–3.64, *p* = 0.029) were also independently associated with suboptimal neurodevelopmental performance.

**Table 4:** Multivariable logistic regression for a Total Gesell Score < 85.

Variable	OR	95% CI	*p*-value
Maternal serum retinol, trimester-averaged (μmol/L)	0.02	0.01–1.27	0.238
Maternal serum vitamin K, trimester-averaged (ng/mL)	1.15	0.16–8.19	0.260
Maternal serum ferritin, trimester-averaged (per 10 ng/mL)	1.22	1.004–1.34	0.036
Maternal age (years)	1.06	1.01–1.13	0.049
Pre-pregnancy BMI (kg/m^2^)	0.99	0.92–1.07	0.832
Years of formal education, years	0.90	0.82–1.00	0.055
Gestational age at delivery (weeks)	0.80	0.64–0.99	0.039
Infant sex (male)	1.98	1.07–3.64	0.029
Maternal HbA1c (%)	1.23	0.69–2.20	0.488

Fully adjusted model included all variables shown. Exact confidence limits are reported with sufficient decimal precision to avoid inconsistencies created by rounding. OR, odds ratio; CI, confidence interval; BMI, body mass index; HbA1c, glycated hemoglobin.

In sensitivity analyses using GDM status instead of maternal HbA1c in the logistic regression models, the association between ferritin and suboptimal neurodevelopmental performance was attenuated and no longer statistically significant (OR = 1.014 per 10 ng/mL, 95% CI: 0.996–1.032, *p* = 0.132; Supplementary Table S2). In contrast, GDM status itself was not independently associated with the outcome in this model (OR = 1.49, 95% CI: 0.58–3.83, *p* = 0.411). In linear regression using GDM status instead of HbA1c, ferritin was not significantly associated with Total Gesell Score (β = −0.019, 95% CI: −0.054 to 0.017, *p* = 0.299), whereas GDM status was strongly associated with lower Total Gesell Score (β = −4.51, 95% CI: −6.70 to −2.32, *p* < 0.001).

### Dose–response relationship with nutritional risk score

The composite Nutritional Risk Score (range 0–3, reflecting lowest-quartile serum retinol, lowest-quartile serum vitamin K, and highest-quartile serum ferritin) was examined in a separate logistic regression model (Table 5). Compared with a score of 0, a score of 1 was associated with a nearly five-fold increased odds of suboptimal neurodevelopmental performance (OR = 4.92, 95% CI: 1.65–14.64, *p* = 0.004). Scores of 2 and 3 also showed elevated odds (OR = 1.43 and 2.66, respectively), but the confidence intervals included unity and the *p*-values were not statistically significant (*p* = 0.433 and *p* = 0.246). Predicted probabilities of suboptimal neurodevelopmental performance derived from the model were 64.3% for a score of 0, 88.9% for a score of 1, 71.4% for a score of 2, and 81.7% for a score of 3.

**Table 5:** Exploratory association between the composite biomarker risk score and Total Gesell Score < 85.

Risk score	*n* (%)	OR	95% CI	*p*-value
0 (ref)	131 (54.6%)	1.00	–	–
1	50 (20.8%)	4.92	1.65–14.64	0.004
2	47 (19.6%)	1.43	0.59–3.45	0.433
3	12 (5.0%)	2.66	0.51–13.84	0.246

Adjusted for maternal age, pre-pregnancy BMI, years of formal education, gestational age at delivery, infant sex, and maternal HbA1c. The composite score ranged from 0 to 3 and assigned one point for each sample-specific biomarker category: serum retinol in the lowest quartile ( ≤ 0.35 μmol/L), serum vitamin K in the lowest quartile ( ≤ 0.57 ng/mL), and serum ferritin in the highest quartile (≥120.43 ng/mL). These categories are exploratory distribution-based classifications and are not clinical definitions of deficiency or excess. These cutoffs were derived from the distribution of trimester-averaged biomarker values in the full cohort and should be interpreted as exploratory sample-distribution thresholds rather than clinical diagnostic criteria.

### Linear regression for Total Gesell Score

Multivariable linear regression was performed to assess the relationship between mean trimester-averaged maternal serum biomarker concentrations and the continuous Total Gesell Score (Table 6). After adjusting for maternal age, pre-pregnancy BMI, education, gestational age, infant sex, maternal HbA1c, CRP, and IL-6, higher mean serum ferritin remained significantly associated with lower Total Gesell Score (β = −0.05, SE = 0.02, *p* = 0.003). No significant associations were observed for mean serum retinol (β = 9.41, *p* = 0.148), mean serum vitamin K (β = 6.72, *p* = 0.132), or IL-6 (β = −0.37, *p* = 0.090). The overall model explained 28.3% of the variance in Total Gesell Score [R^2^ = 0.283, adjusted R^2^ = 0.249, F (11, 228) = 8.19, *p* < 0.001].

**Table 6:** Multivariable linear regression for Total Gesell Score at 12 months.

Variable	β (coefficient)	95% CI	*p*-value
Maternal serum retinol, trimester-averaged (μmol/L)	9.41	−3.37 to 22.19	0.148
Maternal serum vitamin K, trimester-averaged (ng/mL)	6.72	−2.03 to 15.48	0.132
Maternal serum ferritin, trimester-averaged (ng/mL)	−0.05	−0.08 to −0.02	0.003
Maternal age (years)	−0.09	−0.21 to 0.03	0.139
Pre-pregnancy BMI (kg/m^2^)	0.01	−0.14 to 0.16	0.864
Years of formal education	0.27	0.07 to 0.47	0.008
Gestational age at delivery (weeks)	0.09	−0.32 to 0.50	0.657
Infant sex (male)	−0.55	−1.76 to 0.67	0.377
Maternal HbA1c (%)	−0.39	−1.51 to 0.73	0.494
Maternal CRP (mg/L)	−0.05	−0.29 to 0.20	0.713
Maternal IL-6 (pg/mL)	−0.37	−0.80 to 0.06	0.090

Model fit: R^2^ = 0.283, adjusted R^2^ = 0.249, F (11, 228) = 8.19, *p* < 0.001. β values are unstandardized regression coefficients; SE, standard error; BMI, body mass index; CRP, C-reactive protein; HbA1c, glycated hemoglobin; IL-6, interleukin-6.

In sensitivity analyses using CRP-adjusted ferritin values, the association between ferritin and suboptimal neurodevelopmental performance remained statistically significant. In logistic regression, each 10 ng/mL increase in CRP-adjusted ferritin was associated with increased odds of suboptimal neurodevelopmental performance (OR = 1.34, 95% CI: 1.18–1.52, *p* < 0.001). In linear regression, CRP-adjusted ferritin remained significantly associated with lower Total Gesell Score (β = −0.12, SE = 0.01, *p* < 0.001). Raw and adjusted ferritin were highly correlated (*r* = 0.988). These findings suggest that the association between ferritin and suboptimal neurodevelopmental performance is not fully explained by inflammation alone, though residual confounding by unmeasured inflammatory markers cannot be excluded (Supplementary Table S3). In exploratory mediation analyses, neither birth weight nor Apgar score significantly mediated the association between GDM or ferritin and offspring neurodevelopment (all indirect effects *p* > 0.05; Supplementary Table S4).

In exploratory interaction analyses, the Ferritin × GDM interaction term was not statistically significant in either the logistic model for suboptimal neurodevelopmental performance (OR = 1.03, 95% CI: 0.99–1.07, *p* = 0.170) or the linear model for continuous Total Gesell Score (β = −0.01, 95% CI: −0.08 to 0.06, *p* = 0.786; Supplementary Table S5). These findings suggest that the association between maternal serum ferritin and offspring neurodevelopment did not differ significantly by GDM status. However, these analyses were exploratory and limited by sample size.

## Discussion

In this prospective case-control study of 240 mother-infant pairs, we observed that women with GDM had significantly lower serum vitamin A and vitamin K concentrations and higher ferritin levels across all three trimesters compared with control participants. Offspring of participants with GDM exhibited poorer neurodevelopmental outcomes at 12 months, with lower scores across all five Gesell domains and a substantially higher prevalence of Total Gesell Score < 85. Although the between-group differences in mean Gesell domain and total scores were statistically significant, the group means remained within broadly similar interpretive ranges. These differences should therefore not be interpreted as demonstrating a categorical clinical shift for the average child. The findings are more appropriately interpreted as evidence of a difference in the distribution of developmental performance, particularly in the proportion of offspring meeting the study-defined operational threshold for suboptimal neurodevelopmental performance. Because the < 85 threshold includes borderline scores, these prevalence estimates should not be equated with clinically diagnosed developmental delay. After adjusting for potential confounders, only higher mean ferritin level remained independently associated with increased odds of suboptimal neurodevelopmental performance, whereas vitamin A and vitamin K did not reach statistical significance in the fully adjusted logistic model. However, a composite Nutritional Risk Score of 1 (reflecting any one of the three risk conditions) was associated with a nearly five-fold increased odds of suboptimal neurodevelopmental performance, suggesting that the presence of one biomarker-risk category was associated with higher odds of the study-defined neurodevelopmental outcome; however, this finding should be considered exploratory given the non-monotonic pattern across higher scores. Linear regression analyses confirmed that higher ferritin was significantly associated with lower Total Gesell Score, while vitamin A and K showed positive but non-significant coefficients.

First-trimester dietary intake data provide important context for interpreting the observed biomarker differences. Dietary intake was assessed using a semi-quantitative FFQ that has been evaluated for reproducibility and relative validity in pregnant Chinese women (34). Nevertheless, FFQ-based assessment remains subject to recall error, systematic under- or over-reporting, and measurement error. Therefore, the observed relationships between dietary intake and circulating biomarkers should be interpreted as supportive rather than definitive evidence of dietary causation. Participants with GDM had significantly lower dietary vitamin A and vitamin K intake compared with control participants, which is consistent with their lower serum retinol and serum vitamin K concentrations. These findings suggest that lower dietary consumption of fat-soluble vitamins may contribute to the observed reductions in circulating vitamin A and K levels in participants with GDM. Additionally, participants with GDM reported substantially lower intake of fruits and vegetables and lower fiber intake, which are rich sources of these vitamins. The GDM group also had higher energy, carbohydrate, and fat intake, and lower protein intake, suggesting a dietary pattern associated with metabolic dysregulation. In contrast, dietary iron intake did not differ significantly between groups, yet serum ferritin levels were markedly higher in the GDM group across all trimesters. The discordance between dietary iron intake and serum ferritin concentrations may be consistent with higher systemic inflammation, altered iron storage or sequestration, metabolic dysfunction, or a combination of these processes.

The finding that GDM is associated with lower vitamin A and vitamin K levels throughout pregnancy is consistent with previous reports of fat-soluble vitamin deficiencies in diabetic and hyperglycemic pregnancies. Vitamin A plays an essential role in neuronal differentiation, synaptic plasticity, and visual system development, whereas vitamin K is involved in sphingolipid synthesis and myelination (6, 11, 14, 23, 48). The sustained reductions we observed across all three trimesters suggest that GDM may exert a chronic effect on maternal fat-soluble vitamin status, potentially through altered intestinal absorption, increased urinary excretion, or enhanced metabolic utilization (18, 49). Despite these clear group differences in unadjusted analyses, vitamin A and K did not remain independently associated with suboptimal neurodevelopmental performance after adjustment for multiple covariates, including maternal HbA1c, inflammatory markers, and sociodemographic factors. This may indicate that the effect of these vitamins on neurodevelopment is partly mediated by glycemic control or inflammation, or that the null results reflect a true lack of association. Alternatively, our study may have been underpowered to detect smaller effect sizes given the wide confidence intervals observed (7, 50, 51).

The elevated serum ferritin concentrations in women with GDM warrant careful interpretation. Ferritin is both an iron storage protein and an acute-phase reactant (16). The GDM group had higher CRP and IL-6 levels, suggestive of chronic low-grade inflammation (1, 52). Dietary iron intake did not differ significantly between groups, suggesting that the higher serum ferritin concentrations are not explained by dietary intake alone. Ferritin alone cannot establish iron storage, functional iron excess, or iron overload (53). Higher serum ferritin was associated with suboptimal neurodevelopmental performance; however, this association should not be interpreted causally. Ferritin may be a marker of inflammation, metabolic dysfunction, or other unmeasured factors rather than a direct causal exposure (13, 26, 54, 55). Although maternal CRP and IL-6 were included in the linear regression model, they were not included in the primary logistic regression model. Mechanistically, elevated serum ferritin may also reflect iron overload or dysregulated iron metabolism that can promote oxidative stress (16). Oxidative stress may in turn contribute to adverse neurodevelopmental outcomes, providing a plausible hypothetical mechanism linking elevated maternal ferritin to impaired neurodevelopment (56). Alternatively, serum ferritin may simply mark underlying metabolic dysfunction not fully captured by our covariates (13, 16, 20). Future studies incorporating hepcidin, transferrin saturation, and oxidative-stress markers are needed to clarify these pathways. The Nutritional Risk Score was exploratory and did not demonstrate a monotonic dose-response relationship. A score of 1 was associated with increased odds of suboptimal neurodevelopmental performance (OR = 4.92), whereas scores of 2 and 3 showed elevated but non-significant odds ratios with wider confidence intervals, likely reflecting the small number of women with scores of 2 or 3. Importantly, the Nutritional Risk Score did not provide evidence beyond the continuous biomarker models; the results of those models suggest that ferritin is the primary biomarker driving the association, and the composite score does not appear to add unique predictive information. The lack of a clear increasing trend beyond score 1 may also indicate that the three risk components are not simply additive in their effects, or that the optimal cutoffs for defining “low” and “high” require further refinement. In exploratory interaction analyses, we found no statistically significant interaction between GDM status and ferritin on the odds of suboptimal neurodevelopmental performance (*p* = 0.170; Supplementary Table S5), suggesting that the association between ferritin and neurodevelopment did not differ significantly by GDM status. However, these analyses were exploratory and limited by sample size.

Several strengths of this study deserve mention. The prospective design with trimester-specific repeated biomarker measurements provided descriptive information on biomarker concentrations across pregnancy. Non-significant group-by-time interactions (Supplementary Table S1) supported the use of trimester-averaged concentrations in primary analyses. However, the averaging approach did not model within-individual trajectories; trimester-specific analyses would be needed to assess the timing of biomarker changes. The use of a validated and standardized neurodevelopmental assessment tool (Gesell Developmental Schedules) with Chinese norms, administered by blinded assessors, minimized measurement bias. The construction of a composite Nutritional Risk Score—using quartile-defined cutoffs for serum retinol in the lowest quartile ( ≤ 0.35 μmol/L), serum vitamin K in the lowest quartile ( ≤ 0.57 ng/mL), and serum ferritin in the highest quartile (≥120.43 ng/mL)—provides an exploratory, sample-specific composite classification. However, we acknowledge that these cutoffs are data-driven and population-specific rather than based on established clinical thresholds. External validation is needed to determine whether the specific sample-derived thresholds and the resulting composite score are reproducible in independent pregnancy cohorts and whether they are associated with clinically meaningful outcomes. The general use of quartile categorization is a statistical method; however, the clinical relevance of the particular thresholds identified in this study has not been established. The sample size of 240 participants was calculated to detect a 6-point difference in the continuous Total Gesell Score between GDM and control groups, which is consistent with previously reported differences in neurodevelopmental outcomes between offspring of mothers with and without GDM (6, 26). This difference is clinically meaningful as it corresponds to approximately half a standard deviation on the Gesell scale and may affect the proportion of children falling below diagnostic thresholds. Although the study was not specifically powered for the multivariable associations between individual biomarkers and the binary < 85 outcome, the 87 events (children with Total Gesell Score < 85) provide sufficient statistical power for the logistic regression models, which included 6–8 covariates and satisfied the recommended events-per-variable ratio of at least 10.

Limitations must also be acknowledged. First, the observational design precludes causal inference; we can only report associations. Second, residual confounding by unmeasured factors such as polymorphism cannot be excluded. Third, the generalizability of our findings may be limited to populations similar to our study setting. Fourth, the wide confidence intervals for vitamin A and K in regression models suggest imprecise estimates, possibly due to collinearity or limited sample size for subgroup analyses. Fifth, serum ferritin interpretation is complicated by its dual role as an iron-related biomarker and acute-phase reactant (7, 10). Maternal CRP and maternal IL-6 were included in the linear regression model, but not in the primary logistic regression model. In addition, AGP was unavailable, precluding full BRINDA adjustment. CRP-adjusted ferritin sensitivity analyses remained significant (logistic OR = 1.34, 95% CI: 1.18–1.52, *p* < 0.001; linear β = −0.12, *p* < 0.001), consistent with primary findings (Supplementary Table S2). However, CRP-based correction is not equivalent to full BRINDA adjustment, and residual confounding may remain. Thus, ferritin's association with neurodevelopmental outcomes should be interpreted cautiously, as it may reflect both iron-related physiology and inflammation. Additionally, maternal CRP and maternal IL-6 were included only in the linear regression model, and other complementary indicators of iron status, such as transferrin, transferrin saturation, and hepcidin, were not measured. Therefore, we cannot determine whether elevated **serum** ferritin in the GDM group reflects true iron overload, inflammation-driven ferritin elevation, or a combination of both. This limitation should be considered when interpreting the association between ferritin and neurodevelopmental outcomes. Sixth, the neurodevelopmental assessment at 12 months' captures only early milestones; longer-term follow-up is needed. Finally, the high prevalence of Total Gesell Score < 85 in the GDM group (72.5%) and overall cohort (52.1%) warrants careful interpretation. Because this threshold includes borderline scores (75–85) (21–24), the prevalence estimates should be interpreted as an operational classification for early identification and surveillance rather than a clinical diagnosis. Nevertheless, the substantially higher prevalence in the GDM group compared with controls (72.5% vs. 31.7%) underscores the potential impact of GDM-related metabolic and inflammatory factors on early neurodevelopment (21–24). Future studies using different thresholds and longer-term follow-up with validated age-appropriate assessments would help clarify the clinical significance of these findings. We acknowledge that the < 75 threshold has been suggested to identify children at true risk for developmental delay; however, the number of children in our cohort with a Total Gesell Score < 75 was small, particularly in the control group. With the prespecified covariates in the logistic model, the events-per-variable ratio was below the recommended minimum. Therefore, we did not perform additional analyses using the < 75 threshold because of limited statistical power, and we state this limitation directly.

### Implications for clinical practice and future research

Despite these limitations, our findings suggest that maternal ferritin levels in participants with GDM may be associated with offspring neurodevelopmental performance. Given that ferritin is already measured routinely in many antenatal settings, its potential utility for risk stratification remains to be established in independent cohorts. However, we emphasize that the < 85 threshold used in this study was an operational definition for early identification and surveillance and should not be interpreted as a clinical diagnosis. Future research should examine whether different thresholds (e.g., < 75 or < 80) yield similar associations and whether the findings are replicated in other populations with different normative frameworks. The composite exploratory composite Nutritional Risk Score, particularly a score of 1 or higher, may provide a hypothesis-generating framework for identifying biomarker cutoffs that warrant further investigation. Future randomized controlled trials should test whether iron management strategies–such as avoiding unnecessary iron supplementation in women with adequate or elevated ferritin–can improve neurodevelopmental outcomes.

## Conclusions

In this study, participants with GDM had lower maternal serum retinol and serum vitamin K concentrations and higher maternal serum ferritin concentrations across all three trimesters compared with control participants. Higher maternal serum ferritin was associated with higher odds of a Total Gesell Score < 85 (using a screening-oriented threshold) and lower continuous Total Gesell Scores, whereas serum retinol and serum vitamin K showed no statistically significant independent associations in the fully adjusted models. The exploratory composite Nutritional Risk Score was associated with the outcome for a score of 1, but the association was not monotonic across higher score categories. Because serum ferritin is influenced by inflammation, complementary iron-status measures were unavailable, and the composite score used sample-specific quartile thresholds, these findings should be interpreted as hypothesis-generating rather than as evidence for clinical risk stratification or treatment. The specific thresholds identified in this study require external validation in independent cohorts before they can be considered for clinical application.

## Data Availability

The original contributions presented in the study are included in the article/Supplementary material, further inquiries can be directed to the corresponding author.
