# Supplementary material for: Maternal serum retinol, vitamin K, and ferritin profiles during pregnancy, gestational diabetes mellitus, and offspring neurodevelopment: a prospective case-control study
Source: Front Nutr. 2026 Oct 23;13:1967646. doi: 10.3389/fnut.2026.1967646 (PMC13739549; doi:10.3389/fnut.2026.1967646)
Supplement: Supplementary file 1 [file Table_1.docx]

**Supplementary Table**

**Supplementary Table S1. Maternal Serum Biomarker Concentrations Across Trimesters by GDM Status and Repeated Measures ANOVA Results**

| Biomarker | Group | Trimester 1 Mean ± SD | Trimester 2 Mean ± SD | Trimester 3 Mean ± SD | Group×Time χ² | p-value |
| --- | --- | --- | --- | --- | --- | --- |
| Serum Ferritin (ng/mL) | Control (n=120) | 77.33 ± 26.74 | 80.70 ± 28.57 | 83.37 ± 28.39 | 3.36 | 0.186 |
|  | GDM (n=120) | 122.95 ± 30.85 | 118.97 ± 29.79 | 119.70 ± 31.45 |  |  |
| Serum Retinol (μmol/L) | Control (n=120) | 0.45 ± 0.08 | 0.45 ± 0.08 | 0.45 ± 0.07 | 1.32 | 0.517 |
|  | GDM (n=120) | 0.34 ± 0.08 | 0.36 ± 0.08 | 0.36 ± 0.08 |  |  |
| Serum Vitamin K (ng/mL) | Control (n=120) | 0.72 ± 0.10 | 0.70 ± 0.11 | 0.72 ± 0.10 | 2.78 | 0.249 |
|  | GDM (n=120) | 0.57 ± 0.09 | 0.57 ± 0.10 | 0.56 ± 0.10 |  |  |

**Table Note:** Values are presented as mean ± standard deviation. Repeated measures ANOVA was performed with group (GDM vs. control) as the between-subjects factor and trimester as the within-subjects factor. The group-by-time interaction was tested for each biomarker using Linear mixed-effects models with participant-level random intercepts were used to assess group-by-trimester interactions. for participants. T1, first trimester (8–12 weeks); T2, second trimester (24–28 weeks); T3, third trimester (32–36 weeks).

**Supplementary Table S2. Sensitivity Analysis Using GDM Status Instead of HbA1c**

| Model | Variable | OR/β | 95% CI | p-value |
| --- | --- | --- | --- | --- |
| **Logistic Regression** |  |  |  |  |
|  | Maternal serum ferritin (per 10 ng/mL) | OR = 1.014 | 0.996–1.032 | 0.132 |
|  | Maternal age (years) | OR = 1.059 | 0.998–1.124 | 0.058 |
|  | Pre-pregnancy BMI (kg/m²) | OR = 0.992 | 0.918–1.072 | 0.831 |
|  | Years of formal education | OR = 0.905 | 0.818–1.002 | 0.054 |
|  | Gestational age at delivery (weeks) | OR = 0.799 | 0.643–0.992 | 0.042 |
|  | Infant sex (male) | OR = 1.864 | 1.023–3.399 | 0.042 |
|  | GDM status (yes/no) | OR = 1.487 | 0.578–3.827 | 0.411 |
| **Linear Regression** |  |  |  |  |
|  | Maternal serum ferritin (ng/mL) | β = -0.019 | -0.054 to 0.017 | 0.299 |
|  | Maternal age (years) | β = -0.077 | -0.192 to 0.038 | 0.190 |
|  | Pre-pregnancy BMI (kg/m²) | β = 0.037 | -0.110 to 0.184 | 0.619 |
|  | Years of formal education | β = 0.267 | 0.075 to 0.459 | 0.007 |
|  | Gestational age at delivery (weeks) | β = 0.052 | -0.347 to 0.450 | 0.798 |
|  | Infant sex (male) | β = -0.424 | -1.603 to 0.754 | 0.479 |
|  | GDM status (yes/no) | β = -4.509 | -6.696 to -2.323 | <0.001 |
|  | Maternal CRP (mg/L) | β = 0.015 | -0.230 to 0.260 | 0.905 |
|  | Maternal IL-6 (pg/mL) | β = -0.169 | -0.608 to 0.270 | 0.449 |

***Table Note:*** *All models adjusted for maternal age, pre-pregnancy BMI, years of formal education, gestational age at delivery, infant sex, and GDM status (instead of maternal HbA1c). Linear regression additionally included maternal CRP and IL-6. OR, odds ratio; CI, confidence interval; β, unstandardized regression coefficient; BMI, body mass index; GDM, gestational diabetes mellitus; CRP, C-reactive protein; IL-6, interleukin-6.*

**Supplementary Table S3. Comparison of Raw and CRP-Adjusted Ferritin in Logistic and Linear Regression Models**

| **Model** | **Dependent Variable** | **Ferritin Variable** | **OR/β** | **95% CI** | **p-value** |
| --- | --- | --- | --- | --- | --- |
| **Logistic Regression** | Total Gesell Score <85 |  |  |  |  |
| Model 1 |  | Raw ferritin (per 10 ng/mL) | OR = 1.18 | 1.01–1.39 | 0.036 |
| Model 2 |  | CRP-adjusted ferritin (per 10 ng/mL) | OR = 1.22 | 1.04–1.44 | 0.017 |
| **Linear Regression** | Total Gesell Score |  |  |  |  |
| Model 1 |  | Raw ferritin (ng/mL) | β = −0.047 | −0.08 to −0.02 | 0.003 |
| Model 2 |  | CRP-adjusted ferritin (ng/mL) | β = −0.052 | −0.08 to −0.02 | 0.001 |

***Table Note.*** *All models were adjusted for maternal age, pre-pregnancy BMI, years of formal education, gestational age at delivery, infant sex, and maternal HbA1c.* ***Both Model 1 and Model 2 also included serum retinol and serum vitamin K as covariates.*** *Linear regression models additionally included maternal CRP and IL-6. CRP-adjusted ferritin was calculated using an adaptation of the BRINDA regression correction approach [47]. CRP-adjusted ferritin models included 220 women (20 women had missing CRP values); raw ferritin models included all 240 women. OR, odds ratio; CI, confidence interval; β, unstandardized regression coefficient; BMI, body mass index; CRP, C-reactive protein; HbA1c, glycated hemoglobin; IL-6, interleukin-6.*

**Supplementary Table S4. Exploratory Mediation Analysis: Birth Weight and Apgar Score as Potential Mediators of the Relationship Between GDM and Offspring Neurodevelopment**

**Panel A. Binary Outcome: Suboptimal Neurodevelopmental Performance (Total Gesell Score <85) Using the Baron and Kenny Approach**

| Step | Model | Estimate | 95% CI | p-value |
| --- | --- | --- | --- | --- |
| **Mediator: Birth Weight** |  |  |  |  |
| 1 | GDM → Neurodevelopmental Delay (total effect) | OR = 2.35 | 1.05–5.24 | 0.037 |
| 2 | GDM → Birth Weight | β = −75.11 g | −200.52 to 50.31 | 0.239 |
| 3 | Birth Weight → Neurodevelopmental Delay (adjusting for GDM) | OR = 0.9995 per g | 0.9987–1.0003 | 0.228 |
| 4 | GDM → Neurodevelopmental Delay (adjusting for Birth Weight) | OR = 2.28 | 1.02–5.11 | 0.045 |
|  | **Conclusion** | Mediation criteria not met |  |  |
| **Mediator: Apgar Score** |  |  |  |  |
| 1 | GDM → Neurodevelopmental Delay (total effect) | OR = 2.35 | 1.05–5.24 | 0.037 |
| 2 | GDM → Apgar Score | β = −0.38 | −0.52 to −0.24 | <0.001 |
| 3 | Apgar Score → Neurodevelopmental Delay (adjusting for GDM) | OR = 0.68 | 0.36–1.28 | 0.231 |
| 4 | GDM → Neurodevelopmental Delay (adjusting for Apgar Score) | OR = 2.12 | 0.93–4.83 | 0.073 |
|  | **Conclusion** | Mediation criteria not met |  |  |

**Panel B. Continuous Outcome: Total Gesell Score — Indirect Effects**

| Pathway | Indirect Effect (β) | 95% CI | p-value |
| --- | --- | --- | --- |
| GDM → Birth Weight → Total Gesell Score | −0.12 | −0.28 to 0.04 | 0.142 |
| GDM → Apgar Score → Total Gesell Score | −0.08 | −0.22 to 0.06 | 0.261 |
| Ferritin → Birth Weight → Total Gesell Score | −0.03 | −0.09 to 0.03 | 0.328 |
| Ferritin → Apgar Score → Total Gesell Score | −0.02 | −0.07 to 0.03 | 0.432 |

***Table Note.*** *All models were adjusted for maternal age, pre-pregnancy BMI, years of formal education, gestational age at delivery, infant sex, and maternal HbA1c. Linear regression models for the continuous outcome additionally included maternal CRP and IL-6. Panel A uses the Baron and Kenny approach to examine mediation of the binary outcome (Total Gesell Score <85). For birth weight, GDM did not significantly predict birth weight (Step 2, p = 0.239), and birth weight did not significantly predict the outcome after adjustment for GDM (Step 3, p = 0.228), indicating that mediation criteria were not met. For Apgar score, although GDM significantly predicted Apgar score (Step 2, p < 0.001), Apgar score did not significantly predict the outcome after adjustment for GDM (Step 3, p = 0.231), again indicating that mediation criteria were not met. Panel B presents indirect effects for the continuous Total Gesell Score; none of the indirect effects were statistically significant. These exploratory findings suggest that birth weight and Apgar score do not mediate the relationship between GDM (or ferritin) and offspring neurodevelopment in this cohort. Consequently, their exclusion from the primary adjustment set is justified, as including them would not alter the interpretation of the direct effects and could introduce overadjustment bias. BMI, body mass index; CI, confidence interval; CRP, C-reactive protein; GDM, gestational diabetes mellitus; HbA1c, glycated hemoglobin; IL-6, interleukin-6; OR, odds ratio.*

**Supplementary Table S5. Exploratory Interaction Analysis: GDM Status × Ferritin**

| Model | Term | OR/β | 95% CI | p-value |
| --- | --- | --- | --- | --- |
| **Logistic** (Outcome: Total Gesell Score <85) | Ferritin (per 1 ng/mL) | 1.00 | 0.98–1.03 | 0.856 |
|  | GDM status (yes/no) | 0.09 | 0.002–4.93 | 0.242 |
|  | **Ferritin × GDM** | **1.03** | **0.99–1.07** | **0.170** |
| **Linear** (Outcome: Total Gesell Score) | Ferritin (per 1 ng/mL) | −0.01 | −0.07 to 0.04 | 0.620 |
|  | GDM status (yes/no) | −3.65 | −11.16 to 3.85 | 0.338 |
|  | **Ferritin × GDM** | **−0.01** | **−0.08 to 0.06** | **0.786** |

***Table Note.*** *All models adjusted for maternal age, pre-pregnancy BMI, years of formal education, gestational age at delivery, infant sex, and maternal HbA1c. Linear regression additionally included maternal CRP and IL-6. The interaction term represents the multiplicative effect of GDM status on the ferritin–neurodevelopment association. A non-significant interaction indicates that the association between ferritin and neurodevelopment did not differ significantly by GDM status. OR, odds ratio; CI, confidence interval; β, unstandardized regression coefficient; BMI, body mass index; GDM, gestational diabetes mellitus; CRP, C-reactive protein; IL-6, interleukin-6.*
